# Transcript profiling of candidate genes in testis of pigs exhibiting large differences in androstenone levels

**DOI:** 10.1186/1471-2156-11-4

**Published:** 2010-01-25

**Authors:** Eli Grindflek, Ingunn Berget, Maren Moe, Paul Oeth, Sigbjørn Lien

**Affiliations:** 1 NORSVIN (The Norwegian Pig Breeders Association), Hamar, Norway; 2 Centre for Integrative Genetics (CIGENE), Norwegian University of Life Sciences, Ås, Norway; 3 Research and Development, Sequenom, Inc., San Diego, CA. 92121, USA; 4 Department of Animal and Aquacultural Sciences, Norwegian University of Life Sciences, Ås, Norway

## Abstract

**Background:**

Boar taint is an unpleasant odor and flavor of the meat and occurs in a high proportion of uncastrated male pigs. Androstenone, a steroid produced in testis and acting as a sex pheromone regulating reproductive function in female pigs, is one of the main compounds responsible for boar taint. The primary goal of the present investigation was to determine the differential gene expression of selected candidate genes related to levels of androstenone in pigs.

**Results:**

Altogether 2560 boars from the Norwegian Landrace and Duroc populations were included in this study. Testicle samples from the 192 boars with most extreme high or low levels of androstenone in fat were used for RNA extraction, and 15 candidate genes were selected and analyzed by real-competitive PCR analysis. The genes Cytochrome P450 c17 (*CYP17A1*), Steroidogenic acute regulatory protein (*STAR*), Aldo-keto reductase family 1 member C4 (*AKR1C4*), Short-chain dehydrogenase/reductase family member 4 (*DHRS4*), Ferritin light polypeptide (*FTL*), Sulfotransferase family 2A, dehydroepiandrosterone-preferring member 1 (*SULT2A1*), Cytochrome P450 subfamily XIA polypeptide 1 (*CYP11A1*), Cytochrome b5 (*CYB5A*), and 17-beta-Hydroxysteroid dehydrogenase IV (*HSD17B4*) were all found to be significantly (P < 0.05) up-regulated in high androstenone boars in both Duroc and Landrace. Furthermore, Cytochrome P450 c19A2 (*CYP19A2*) was down-regulated and progesterone receptor membrane component 1 (*PGRMC1*) was up-regulated in high-androstenone Duroc boars only, while *CYP21 *was significantly down-regulated (2.5) in high-androstenone Landrace only. The genes Nuclear Receptor co-activator 4 (*NCOA4*), Sphingomyrlin phosphodiesterase 1 (*SMPD1*) and 3β-hydroxysteroid dehydrogenase (*HSD3B*) were not significantly differentially expressed in any breeds. Additionally, association studies were performed for the genes with one or more detected SNPs. Association between SNP and androstenone level was observed in *CYB5A *only, suggesting cis-regulation of the differential transcription in this gene.

**Conclusion:**

A large pig material of highly extreme androstenone levels is investigated. The current study contributes to the knowledge about which genes that is differentially expressed regard to the levels of androstenone in pigs. Results in this paper suggest that several genes are important in the regulation of androstenone level in boars and warrant further evaluation of the above mentioned candidate genes, including analyses in different breeds, identification of causal mutations and possible gene interactions.

## Background

Most male pigs used for pork production are castrated very early in life in order to prevent boar taint in the meat. Boar taint is an off-odor/off-flavor primarily caused by high levels of the two compounds androstenone and skatole [1]. Castration is, however, undesirable due to ethical and economical concerns, and because it removes the source of natural anabolic androgens stimulating lean growth. Androstenone is a natural steroid produced by the Leydig cells of the testis in parallel with anabolic hormones [2], and acts as an active sex pheromone regulating reproductive function in female pigs. Due to its lipophilic nature, some androstenone is accumulated in the adipose tissue and produces taint when the fat is heated [3]. Genetics has a large impact on the levels of androstenone in carcass and heritability estimates are found to range from 0.25 to 0.87 [4,5].

Several studies have aimed at finding the enzymes or key regulatory proteins involved in regulation of androstenone concentrations (reviewed by Robic et al. [6], and Zamaratskaia and Squires [7]), although on a limited number of genes. The primary objective of this study was to test for differential expression in fifteen selected candidate genes involved in the regulation of androstenone levels in pigs. Some of the genes were selected as candidate genes for boar taint due to their biological function, while others were selected on the basis of an earlier microarray gene expression study [8]. The latter set of candidate genes were included in order to confirm earlier findings in another and extended animal material. Recent studies have demonstrated the effect of genetic variation on gene expression levels [9,10]. The individual variation in gene expression consists of two varieties; cis-acting which results from DNA variations of a gene that is directly influencing transcription level of that gene, and trans-acting which is due to alteration of other genetic variations. Therefore, we performed an association test examining the phenotypic effects of different alleles for some of the candidate genes (*CYB5A*, *CYP11A1*, *HSD3B *and *NCOA4*) displaying differential expression.

## Results

### Differential gene expression

A total of 12 out of 15 candidate genes were found to be differentially expressed between high/low androstenone groups at a significance level of P < 0.001 to P < 0.05 in one or both of the breeds Landrace and Duroc. All the genes significant differentially expressed (Table 1 and 2) were up-regulated in the high-androstenone boars compared to low-androstenone boars, except for *CYP19A2 *and *CYP21 *which were down-regulated in the high-androstenone boars. The genes Aldo-keto reductase family 1 member C4 (*AKR1C4*), *CYB5A*, Cytochrome P450 c17 (*CYP17*), *CYP11A1*, Short-chain dehydrogenase/reductase family member 4 (*DHRS4*), Ferritin light polypeptide (*FTL*), 17-beta-Hydroxysteroid dehydrogenase IV (*HSD17B4*), Steroidogenic acute regulatory protein (*STAR*) and Sulfotransferase family 2A dehydroepiandrosterone-preferring member 1 (*SULT2A1*) were all significantly up-regulated in high-androstenone Duroc and Landrace boars. Furthermore, Cytochrome P450 c19A2 (*CYP19A2*) was down-regulated and progesterone receptor membrane component 1 (*PGRMC1*) was up-regulated in high-androstenone Duroc boars only, while *CYP21 *was significantly down-regulated (2.5) in high-androstenone Landrace only. The genes *NCOA4*, Sphingomyrlin phosphodiesterase 1 (*SMPD1*) and *HSD3B *were not significantly differentially expressed in any breeds.

**Table 1:** Results from the rcPCR bootstrap statistics (×4000), Landrace.

Gene	Fold change^a^	Log10 Fold change	Bias	Std error	P value
AKR1C4	2.6	0.42	0.0050	0.11	0.0000
CYB5A_-8(5'UTR)	2.6	0.42	0.0034	0.17	0.0090
CYB5A_ iso1-2	2.4	0.37	0.0026	0.19	0.0210
CYP11A1	3.1	0.50	0.0050	0.13	0.0000
CYP17A1	2.9	0.46	0.0034	0.09	0.0000
CYP19A2	0.8	-0.10	-0.0003	0.13	0.2120
CYP21_exon9	0.1	-0.91	-0.0033	0.46	0.0200
DHRS4	2.6	0.41	0.0043	0.11	0.0005
FTL	2.3	0.35	0.0036	0.11	0.0005
HSD3B_exon2	1.2	0.06	0.0067	0.24	0.3810
HSD3B_5'UTR	0.8	-0.10	0.0023	0.20	0.3120
HSD17B4	2.2	0.34	0.0034	0.12	0.0030
NCOA4	1.3	0.11	0.0019	0.13	0.1970
PGRMC1	1.2	0.08	0.0045	0.13	0.2700
SMPD1	1.1	0.04	0.0032	0.09	0.3200
STAR	13.5	1.13	0.0032	0.14	0.0000
SULT2A1	3.0	0.48	0.0031	0.13	0.0002

^a^Fold changes are calculated relative to baseline, which is the group of low androstenone (LL) in this case, and are therefore indicating the times of up-regulation in high-androstenone group compared to the low-androstenone group. All genes are adjusted for the housekeeping gene HPRT.

**Table 2:** Results from the rcPCR bootstrap statistics (×4000), Duroc.

Gene	Fold change^a^	Log10 Fold change	Bias	Std error	P value
AKR1C4	1.6	0.21	0.0007	0.11	0.0270
CYB5A_-8(5'UTR)	2.0	0.31	-0.0011	0.11	0.0040
CYB5A_iso1-2	1.6	0.19	0.0030	0.15	0.0910
CYP11A1	2.3	0.37	0.0006	0.12	0.0010
CYP17A1	2.4	0.38	0.0004	0.11	0.0005
CYP19A2	0.6	-0.22	0.0002	0.12	0.0360
CYP21_exon8	0.9	-0.04	0.0042	0.25	0.4380
CYP21_exon9	1.0	0.01	0.1255	0.32	0.4780
DHRS4	2.1	0.33	0.0011	0.11	0.0020
FTL	1.9	0.27	0.0008	0.11	0.0090
HSD3B_exon2	0.8	-0.11	0.0005	0.17	0.2490
HSD3B_5'UTR	1.0	0.02	-0.0011	0.15	0.4460
HSD17B4	1.6	0.20	0.0002	0.11	0.0430
NCOA4	1.5	0.18	-0.0018	0.11	0.0620
PGRMC1	1.6	0.21	0.0005	0.11	0.0330
SMPD1	1.1	0.03	0.0011	0.11	0.3940
STAR	4.7	0.68	-0.0009	0.12	0.0000
SULT2A1	2.1	0.32	-0.0004	0.11	0.0030

^a^Fold changes are calculated relative to baseline, which is the group of low androstenone (LD) in this case, and are therefore indicating the times of up-regulation in high-androstenone group compared to the low-androstenone group. All genes are adjusted for the housekeeping gene HPRT.

### Allele-specific differential expression

Assays were designed for the investigation of allele-specific differential expression of one SNP within *CYB5A *and two SNPs within each of the genes *CYP21 *and *HSD3B*. Additionally, one assay was made to study differential expression of the *CYB5A *isoforms 1 and 2. Expression of the *CYB5A *isoform 2 was, however, not detected in any of the samples. Also, no significant differential allele-specific expression between high and low androstenone animals (both breeds) was detected in any of the genes investigated (results not shown). For the SNPs in *CYB5A *(-8(5'UTR)), *CYP21 *(exon8 and exon9), as well as for the SNPs in *HSD3B *(-15(5'UTR)), the two alleles had nearly identical expression levels, with expression levels ratios in the range of 0.46-0.54. For the SNP located in *HSD3B *exon2, on the other hand, the two alleles were expressed differentially (P < 0.05), although not with regard to the levels of androstenone. In Duroc, allele A had generally higher expression levels than allele G (average ratio 0.80), whereas in Landrace allele A had lower expression levels than allele G (average ratio 0.41). Notably, allele A is quite rare in both breeds. The allele frequencies used in the assays of allele-specific expression are shown in Table 3.

**Table 3:** Frequency of alleles used in the assays of allele expression, Landrace and Duroc.

Gene, LANDRACE	Genotype	Frequency	Gene, DUROC	Genotype	Frequency
HSD3B_5'UTR	CC	7	HSD3B_5'UTR	CC	4
	CT	34		CT	31
	TT	55		TT	60
HSD3B_exon2	AA	1	HSD3B_exon2	AA	0
	AG	13		AG	16
	GG	82		GG	79
CYP21_exon8	CC	8	CYP21_exon8	CC	55
	CT	32		CT	25
	TT	56		TT	16
CYP21_exon9	CC	64	CYP21_exon9	CC	96
	CT	28		CT	0
	TT	4		TT	0
CYB5A_-8(5'UTR)	GG	94	CYB5A_-8(5'UTR)	GG	89
	GT	2		GT	6
	TT	0		TT	0

### Association studies

Five SNPs were detected in the candidate genes *CYB5A*, *CYP11A1*, *HSD3B *and *NCOA4 *(Table 4), and single SNP association studies were performed in both Landrace and Duroc breeds. The SNP located in position -8 of the *CYB5A *gene was significantly associated to androstenone levels in Duroc (P < 0.01), although it was not reproducible within Landrace (P = 0.14). No other SNPs were significantly associated with androstenone in this study (Table 4). Association results including SNPs in some of the other genes investigated in this study have previously been presented in Moe et al. [11].

**Table 4:** The associations between SNPs and levels of androstenone in Duroc and Landrace boars^a^

B	Gene	Genotype 1	Genotype 1/2	Genotype 2	P value
D	CYB5A_-8(5'UTR)	G(n = 902):3.45 (± 0.12)	G/T(n = 51): 2.56(± 0.38)	-	0.01
D	HSD3B_-15(5'UTR)	C (n = 67): 2.38 (± 0.45)	C/T(n = 293):2.70(± 0.20)	T (n = 409):3.25(± 0.12)	0.11
D	HSD3B_271-exon2	-	A/G(n = 74): 2.82(± 0.11)	G (n = 641):3.03(± 0.28)	0.68
D	NCOA4_3'UTR	A(n = 326):3.48 (± 0.23)	AG(n = 413):3.49(± 0.21)	G(n = 180):3.67 (± 0.26)	0.22
L	CYB5A_-8(5'UTR)	G(n = 1278):1.18(± 0.04)	G/T(n = 25): 0.97 (± 0.15)	-	0.14
L	CYP11A1_150-exo1	A(n = 321):1.05 (± 0.13)	A/G(n = 352):1.23(± 0.11)	G(n = 153):0.96 (± 0.20)	0.39
L	HSD3B_-15(5'UTR)	C(n = 74): 1.00(± 0.19)	C/T(n = 267):1.31(± 0.13)	T(n = 430): 1.02(± 0.11)	0.16
L	HSD3B_271-exon2	A(n = 14): 1.00(± 0.21)	A/G(n = 94): 0.88(± 0.16)	G(n = 512): 1.11(± 0.29)	0.64
L	NCOA4_3'UTR	A(n = 204):1.24(± 0.09)	A/G(n = 530):1.18(± 0.05)	G(n = 425): 1.10(± 0.06)	0.36

^a^The number of boars is shown between parentheses after each genotype. The least square means are shown for all genotypes and estimated standard errors are shown between the parentheses. Dash is no genotype found. B = Breed, D = Duroc and L = Norwegian Landrace.

## Discussion

In the present study, fifteen candidate genes potentially affecting androstenone levels in boars were selected, based on the biochemistry and physiology of the trait, and on results from a microarray study published by Moe et al. [8]. The main objective of the study was to determine whether the genes were differentially expressed in pigs with high and low androstenone levels. Variation in gene expression between different alleles in mammals [12] and genetic variation in single nucleotide polymorphisms (SNPs) within the candidate genes may also contribute to the androstenone variability. Therefore, investigation of allele-specific expression and association tests were also performed for the candidate genes in question.

The synthesis of 16-androstene steroids, including androstenone, occurs by the action of several enzymes and some of them have been found to be more important than others. Members of the Cytochrome P450 superfamily function as monooxygenases, utilizing electrons to catalyze the hydroxylation and cleavage of substrates. The formation of the 16-androstene steroids from pregnenolone is catalyzed by the andien-β synthase enzyme system [13]. Major enzymes in this system are *CYP17A1 *along with *CYB5A *and the associated reductases [14]. Several studies have previously studied *CYB5A *as a candidate gene for boar taint [15-17]. Levels of mRNA for total *CYB5A *were found to be significantly correlated with levels of androstenone in fat [16]. These results are in accordance with our results, showing significant up-regulation of total *CYB5A *(CYB5A_8(5'UTR) in Tables 1 and 2) expression in the high androstenone animals in both breeds. Two variants, a soluble (isoform 1) and membrane bound (isoform 2) form, of *CYB5A *cDNA have been isolated in pigs [18], and later detected as a low molecular weight form (isoform 1) and a high molecular weight form (isoform 2) in porcine testis [16]. The *CYB5A *isoforms are derived from one mRNA via alternative splicing [19]. A study by Davis et al. [16] found that levels of the *CYB5A *isoform 1, but not levels of isoform 2, were correlated with both the rate of 16-androstene steroid synthesis and fat androstenone concentrations, indicating that increased levels of the isoform 1 of *CYB5A *are linked to a higher level of androstenone production in pig testis. In our study, the assay "CYB5A_iso1-2" (Table 1 and 2) was used to study differential expression between isoform 1 and 2. Results show that the expression of isoform 1 was significantly up-regulated (P < 0.05) in high-androstenone Landrace animals. The Duroc breed did not reached the defined level of significance, although it was rather close (P = 0.09). Expression levels of *CYB5A *isoform 1 seems to be slightly less up-regulated (although not significantly) compared with total *CYB5A *expression (Tables 1 and 2). *CYB5A *isoform 2 was not detected in any of the samples. Another purpose of the assay "CYB5A_-8 (5'UTR)" was to study differences in allele-specific expression of the SNP detected in the 5'UTR, 8 bp upstream of the translation start codon. The results revealed no significant differential allele-specific expression between the high and low androstenone groups. Furthermore, an association study on the same polymorphism in the entire material of Landrace and Duroc, i.e. 2560 boars altogether, were performed. Only homozygous G and heterozygous GT animals were detected in our populations, reflecting a low frequency of the T allele which is also seen in other populations [17,20]. The polymorphism was found to be significantly associated to androstenone levels in Duroc, but not in Landrace. In both breeds, however, the LS mean values of androstenone were lower in the heterozygous (GT) animals than in the homozygous G genotype (Table 4). This trend is in accordance with the results of Lin et al. [15] reporting this SNP allele to be associated with a decrease in fat androstenone production *in vivo*, as well as *CYB5A *protein expression in vitro, in a variety of breeds. This is also confirmed by two other studies [17,20]. Both differential gene expression and association with androstenone might indicate a Cis-acting regulation of *CYB5A *expression in pigs. Furthermore, CYB5A is a protein widely involved in biological processes, being a component of electron transfer chains in a number of pathways [21]. For example, interactions between CYB5A and the FTL may affect levels of androstenone through the CYB5A/CYP450 electron transfer [22]. In this study, the *FTL *was highly up-regulated in both breeds (P < 0.01), which is in agreement with the study of Moe et al. [8]. The *FTL *gene provides instructions for making the ferritin light chain. Ferritin stores and releases iron in cells and plays a central role in numerous essential cellular functions (reviewed by Hentze and Kuhn [23]).

The major enzymes Cytochrome P450 c17 (CYP17) and CYB5A interacts in the andien-β synthase system [13], and since CYP17A1 also converts pregnenolone into precursors of the androgens and estrogens it is also a very potent candidate gene for androstenone production. However, no significant effects have so far been detected in association studies [24] or on the protein expression level [16]. In this study we did, however, find *CYP17A1 *cDNA levels to be significantly up-regulated in high androstenone boars of both Landrace and Duroc. No SNPs were detected within the *CYP17A1 *gene in our populations.

The key rate-limiting factor for the maintenance of steroid production is the continuous provision of the cholesterol substrate from the outer mitochondrial membrane to the enzymatic component in the inner membrane, which is mainly facilitated by STAR [25]. Next, the CYP11A1 enzyme, localized to the mitochondrial inner membrane, catalyzes the conversion of cholesterol to pregnenolone in the first and rate-limiting step in the synthesis of the steroid hormones [26]. This is a very important step in the production of androstenone, and interestingly *STAR *and *CYP11A1 *are both highly up-regulated in high androstenone animals in both Landrace and Duroc. *STAR *was found as much as 13.5 times up-regulated in Landrace and 4.7 times up-regulated in Duroc. Highly differentiated expression of *STAR *was also seen in our previous microarray study [8]. *STAR *has previously shown increased gene expression during the time of sexual differentiation [27]. Regulation of STAR has, however, been suggested to be both on the post transcriptional level, in a developmental stage- and tissue-specific manner [28], and at transcription level [29]. No SNPs were detected in *STAR *in this study, and further studies are needed to reveal molecular basis for this variation. Also *CYP11A1 *was found up-regulated in the previous microarray study [8] and confirmed in this study. A SNP located in *CYP11A1 *exon 1 was not significantly associated with androstenone levels in Landrace boars (Table 4), while no data were obtained for the Duroc breed. In contrast, another polymorphism in *CYP11A1 *exon 1 has previously been found to be significantly associated with androstenone levels in Yorkshire boars [30], whilst not in a Large White and Meishan cross [31].

Furthermore, sulfotransferase family 2A dehydroepiandrosterone-preferring member 1 (SULT2A1) is a key enzyme in the testicular and hepatic metabolism of 5α-androstenone and responsible for sulfoconjugating the 16-androstene steroids. Previous studies have indicated that increased levels of sulfoconjugated 16-androstene steroids present in the systemic circulation are associated with reduction in the accumulation of 5α-androstenone in adipose tissue [32]. Additionally, testicular SULT2A1 activity was found to be negatively correlated with 5α-androstenone concentrations in fat, SULT2A1 enzyme activity was positively correlated with SULT2A1 protein level, and finally the gene expression level was positively correlated with increased protein level [33]. The findings in our study are, however, contradictory to this since we have an up-regulation of *SULT2A1 *gene expression level in high androstenone animals (both breeds).

The last steps in the formation of androgens and estrogens are catalyzed by 17β-hydroxysteroid dehydrogenase (17β-HSD) enzymes [34]. Previously, these enzymes have been assigned to porcine Leydig and Sertoli cells [35] and several porcine tissues have been shown to express HSD17B4 as a predominant dehydrogenase [36]. HSD17B4 has also been shown to inactivate estrogens very efficiently in several tissues because of its preference for steroid oxidation [37]. Our study, however, indicate that the *HSD17B4 *gene is rather up-regulated in testes in both high androstenone Duroc and Landrace boars. Due to this it is important to note that several roles of HSD17B4 are suggested [37]. A study done by Chen et al. [38] did not detect any differences in *HSD17B *gene expression between boars of high and low androstenone in a Landrace x Yorkshire crossbred. Five SNPs within HSD17B were detected and tested in this population by Moe et al. [11], but no significant associations were detected.

The gene expression of *AKR1C4*, which belongs to the cytosolic aldo-keto reductases that act as 3α -/3β-/17β-/20α-hydroxysteroid dehydrogenases (HSDs) in human [39], was also investigated. Significant up-regulation of the gene *AKR1C4 *was detected in high androstenone boars in both breeds, although it was more pronounced in Landrace (Tables 1 and 2). This is in accordance with results of Moe et al. [8]. All the isoforms AKR1C1-AKR1C4 have previously been found to convert active androgens and estrogens to their associated inactive metabolites, preventing excess of circulating steroid hormones and turning the steroids into substrates for conjugation reactions [39]. However, the role of AKR1C4 in regulation of androstenone level in testes needs to be clarified. Members of the dehydrogenase/reductase (SDR) family are other enzymes involved in the process of oxidation of 3β-hydroxysteroid precursors into ketosteroids. Several family members have previously been shown to be important in catalyzing an essential step in the biosynthesis of all classes of active steroid hormones [40]. The member *DHRS4 *was found to be highly up-regulated in high androstenone boars in both Landrace and Duroc [8], and this was confirmed in an extended animal material in this study (P < 0.005). Interestingly, the DHRS4 was very recently shown to have a role in 3β-hydroxysteroid synthesis, and DHRS4 was shown to be induced via PPARα activation [41]. PPARα has previously been shown to regulate various genes controlling gluconeogenesis, ketone body synthesis, heme synthesis and cholesterol metabolism [42]

Breed differences in levels of androstenone (e.g. Tajet et al. [5]), sequence variation, mRNA and protein levels have been found in several studies [43-45]. In this study we found breed differences in level of expression for the genes *CYP19A2*, *PGRMC1 *and *CYP21. CYP19A2 *was significantly down-regulated and *PGRMC1 *significantly up-regulated in high androstenone Duroc boars, while none of them were differentially expressed in Landrace. Cytochrome P450 c19 (*CYP19*) encodes the enzyme aromatase, which catalyses the synthesis of estrogens from androgens. Unusually high levels of estrogens are secreted from the porcine testes [46] and pig is the only mammal known to express functionally distinct isoforms of the *CYP19 *gene [47]. Notably, our results for the isoform *CYP19A2 *are not supported by previous microarray results showing up-regulation in high androstenone boars in both breeds [8]. Results in this study are based on more animals compared with the previous microarray study, and results in the current study might suggest that the significant results of differential expressions of CYP19A2 in Moe et al. [8] are false positives. Another explanation might be that other transcripts or isoforms (e.g. CYP19A1, CYP19A3) than CYP19A2 are picked up and quantified in one of the gene expression methods, although the oligo assay designed for CYP19A2 in the rcPCR experiment is made specifically to distinguish between the isoforms. The results for the *PGRMC1 *gene were, however, in concordance with the results reported by Moe et al. [8]. *PGRMC1 *is suggested to have a role in binding heme and to catalyze steroids by cytochrome P450 enzymes, analogous to the roles played by *CYB5A *(reviewed by Cahill [48]). CYP21 is a member of the cytochrome P450 superfamily enzymes, which is a key enzyme for corticosteroidogenesis [49] and suggested to have arisen evolutionary from the same gene as *CYP17A1 *[50]. From a physiological point of view, CYP21 leads to drastic fertility changes in human females [51]. *CYP21 *was significantly down regulated (P < 0.02) in high androstenone Landrace in this study, although it is important to point out that expression levels were generally very low and the standard error high in both breeds (Tables 1 and 2). Gene expression of *CYP21 *in testes has previously not been studied in any species. A QTL for androstenone level of boars from a Large White/Meishan cross was detected in this region, and *CYP21 *was suggested as a positional candidate gene, although no polymorphisms were detected in the coding region and no association study performed [31]. An association study performed on the same populations as described in this paper detected seven SNPs within the *CYP21 *gene, although none of them were significantly associated with androstenone in any of the breeds [11].

The candidate genes *HSD3B*, *NCOA4 *and *SMPD1 *were all chosen because they have relevant functions regarding production of androstenone. HSD3B is an enzyme catalyzing the biosynthesis of steroids in testis [52], and the enzyme has also been shown to catalyze the initial step of the hepatic metabolism of androstenone in pigs [53]. Recently, expression of the 3β-HSD protein was shown to be repressed in liver in pigs with high androstenone, but not in testis [54]. Significantly reduced levels of mRNA expression in high androstenone Landrace and Yorkshire boars were obtained in another study [38]. In this study we were, on the other hand, not able to detect significant differences in gene expression levels of *HSD3B*. Furthermore, no differences in allele expression were observed, as well as none significant associations with any of the SNPs investigated (Table 4). The association results are in concordance with a recent study by Cue et al. [45], obtaining no significant associations between the *HSD3B *SNPs, all located in the 5'UTR, and the androstenone level in fat from several breeds.

Nuclear receptor co-activator 4 (NCOA4; often referred to as ARA70) is identified as an androgen receptor specific co-activator [55], and is suggested to have a role in the modulation of the sex hormone specificity in humans [56]. *NCOA4 *was shown to be significantly up-regulated in high androstenone Duroc boars in the recent microarray study [8]. In this study, however, we were not able to confirm this result in an extended animal material, although results were close to significant (P = 0.06). One SNP from the *NCOA4 *3'UTR region was genotyped in both populations but no significant association was observed (Table 4).

Sphingomyrlin phosphodiesterase 1 (SMPD1) is ubiquitous lysosomal hydrolase that cleaves sphingomyelin to ceramide, which again has been shown to inhibit CYP19 activity through induction of transcription factors [57]. *SMPD1 *was down-regulated in high androstenone Duroc animals in the microarray study [8]. This result was, however, not confirmed in the rcPCR study performed by Moe et al. [8], and not either in the current study performed on extended animal material.

Previous studies have shown that differential expression of alleles is quite common in mammals and that such variation may contribute to phenotypic variability [12,58]. Interestingly, 54% of tested genes were found to have preferential expression of one allele in some individuals and almost half of them showed greater than fourfold difference between the two alleles [12]. Therefore, when possible, assays were designed to allow simultaneous transcript profiling of alleles in a heterozygous individual. Five SNPs in three genes were analyzed to see whether such differentially allelic expression is present, although no significant differences were obtained (results not shown).

Summarizing this study, the genes *AKR1C4, CYB5A, CYP11A1, CYP17A1, CYP19A2, CYP21, DHRS4, FTL, HSD17B4, SULT2A1, STAR *and *PGRMC1 *were found to be differentially expressed in this study. *HSD3B *was not differentially expressed in this study, contradictory to results seen in the Yorkshire breed [38]. Association between SNP and androstenone level was observed in the *CYB5A *gene only, suggesting cis-regulation of differential transcription. The frequency of the favorable allele is, however, very low (see Table 4), which makes it less useful for selection purposes. SNP detection needs to be performed also for the other differentially expressed genes in this study to find potentially useful markers for selection against boar taint. Previous to selection against androstenone it is, however, important also to find the relationship between the candidate SNPs and other reproduction related traits. Two of the most up regulated genes in this study, *STAR *and *CYP17A1*, have for example previously been found to be elevated in preovulatory estrogenic follicles in pigs [59]. Results in this paper suggest that several genes are important in the regulation of androstenone level in boars and warrant further evaluation of the above mentioned candidate genes, including analyses in different breeds, identification of causal mutations and possible gene interactions.

## Conclusion

The gene expression of fifteen candidate genes is investigated in a large pig material of highly extreme androstenone levels. The current study contributes to new knowledge about the genes and pathways involved in regulation of androstenone in pigs, as well as contributing to important confirmation of genes previously investigated. Results highly suggest that several genes are important in the regulation of androstenone level in boars. For some of the genes the results also indicate whether there are cis- or trans regulated differences in level of transcription.

## Methods

### Animals and Sampling

Samples and phenotypes from 1533 Landrace and 1027 Duroc boars were included in this study, and all of them were tested in NORSVIN's (the Norwegian Pig Breeders Association) boar testing stations. The animals were reared on the standard commercial feed with an energy content of 14.9 MJ digestible energy, 17.8% raw protein, 5.6% fiber, 6% raw fat, 6% raw ash and 1.12% lysine, without food or water restrictions. Blood samples were collected from all boars at the boar testing stations up to two weeks before slaughter. All animals were cared for according to laws and internationally recognized guidelines and regulations controlling experiments with live animals in Norway (The Animal Protection Act of December 20th, 1974, and the Animal Protection Ordinance Concerning Experiments with Animals of January 15th, 1996); according to the rules given by Norwegian Animal Research Authority.

The boars were harvested during a period of 26 months and the Landrace and Duroc boars were on average 143 and 156 days at 100 kg live weight, respectively. They were slaughtered 15 days later on average. Samples were taken from testicles on the slaughter line, snap frozen in liquid N_2 _and thereafter stored at -80°C. Blood samples for plasma suspension and DNA extraction were taken three days before slaughter. For androstenone measurements, samples of subcutaneous adipose tissue were collected from the neck region and stored at -20°C. The length of *glandula bulbo urethralis *was measured at the slaughter line. All boars, 2560 altogether, were included in the association study performed for the SNPs detected in 3β-hydroxysteroid dehydrogenases *(HSD3B)*, Cytochrome P450 subfamily XIA polypeptide 1 (*CYP11A1*), Cytochrome b5 *(CYB5A)*, and nuclear receptor co-activator 4 (*NCOA4*), while the 192 boars with most extreme levels of androstenone were selected for gene- and allele expression studies. For gene- and allele expression 6 and 9% of the most extreme animals in Landrace and Duroc were selected, respectively.

### Analyses of Androstenone

The levels of androstenone were analyzed at the hormone laboratory at the Norwegian School of Veterinary Sciences (NVH) by a modified time-resolved fluoroimmunoassay [60], using antibody produced by Andresen [61]. Average androstenone levels were 1.17 μg/g (SD = 1.10) and 3.22 μg/g (SD = 2.69) for the entire Landrace and Duroc populations, respectively.

The 192 most extreme high/low androstenone boars in both Landrace and Duroc were divided into four groups consisting of 48 individuals. The 48 high androstenone Landrace and 48 low androstenone Landrace boars had average androstenone values of 5.62 μg/g (SD = 1.74) and 0.16 μg/g (SD = 0.04), respectively. Likewise, the 48 high androstenone Duroc and 48 low androstenone Duroc boars had average androstenone values of 10.59 μg/g (SD = 2.47) and 0.39 μg/g (SD = 0.14), respectively.

### Nucleic acid purification and cDNA synthesis

Total RNA was isolated from testes using the M48 (Qiagen) and treated with TURBO DNA-free™ (Ambion, Huntingdon, UK) for removal of contaminating DNA. RNA quality and concentration were determined using RNA 6000 Nano LabChip^® ^Kit on 2100 BioAnalyzer (both from Agilent Technologies, USA) and Nanodrop, ND-1000 spectrophotometer (NanoDrop Technologies, DE, USA), respectively. First strand cDNA synthesis was conducted using SuperScript™-II Rnase H^- ^Reverse Transcriptase (Invitrogen, Carlsbad, CA). 0.5 μg of total RNA from each testicle sample was used as template.

DNA used for the association study was isolated from porcine leukocytes using the MagAttract DNA Blood Midi M48 protocol on the Bio-Robot M48 (Qiagen, Hilden, Germany). Concentration and quality were measured on a Nanodrop, ND-1000 spectrophotometer (NanoDrop Technologies, DE, USA) and on a 1420 Victor plate reader (Turku, Finland) using PicoGreen fluorescence (Molecular Probes, OR, USA).

### MassARRAY Assay Design

The porcine gene sequences used to create a multiplexed 19-assay panel for gene- and allele-specific expression analysis via real-competitive PCR (rcPCR) and MassARRAY were annotated with respect to exon/intron boundaries. Assays were designed such that one of the PCR primers spanned an exonic junction (to insure binding specificity to cDNA) using MassARRAY QGE Assay Design software v1.0 (SEQUENOM, San Diego, USA) for all non-polymorphic loci from each transcript. Amplicon sequences from these designs were then used as templates for a second round of assay design to create a multiplex containing the additional polymorphic loci used for allele-specific expression analysis of SNPs located in Cytochrome P450 subfamily 21 (*CYP21*), *HSD3B*, *CYB5A*, and typing of *CYB5A *isoforms. These designs were created using the iQSNP module of the MassARRAY SNP Assay Designer software v3.0 (SEQUENOM, San Diego, USA). Primers and competitors from this design are shown in Additional files 1 and 2.

### Gene expression analysis

Real-competitive (rc) PCR gene expression analysis was used to study differential gene- and allele expression [62]. The method is based on the MassARRAY methodology, using the Quantitative Gene Expression (QGE) iPLEX system (Sequenom, San Diego, CA). The competitor, a synthetic DNA molecule matching the sequence of the targeted cDNA region at all positions except for one single base, served as an internal standard for each transcript. A 10-fold dilution of competitor was initially used over a wide range of concentrations to determine an approximate equivalence point (equal co amplification of target cDNA and competitor), followed by a 7-fold dilution of competitor from 4.04 × 10^-11 ^to 1.43 × 10^-19 ^M (a molar concentration of 1.00 × 10^-18 ^is equivalent to 3 competitor molecules) to achieve more accurate measurements. The cDNA and competitor were co-amplified in the same PCR reaction with PCR conditions 95°C for 15 minutes, followed by 45 cycles of 95°C for 20 second, 56°C for 30 seconds and 72°C for 1 minute, and finally 72°C for 3 minutes. After a clean-up step to remove unincorporated nucleotides, the PCR products were used as templates for the primer extension reaction. The preparation of iPLEX reaction cocktail mix and PCR were performed as described in the Sequenom application guide http://www.sequenom.com/. Parallel PCR-reactions were performed for all samples and each of the products was printed with 2 replicates on a SpectroCHIP. The primer extension reaction generates short oligonucleotides with distinct masses for competitor and cDNA-derived products, and MALDI-TOF mass spectrometric analysis of these DNA fragments generated signals which were quantified based on peak areas for each respective assay. To detect and confirm differentially expressed genes, hypoxanthine guanine phosphoribosyltransferase 1 (*HPRT*) were used as a reference transcript or 'housekeeping gene'.

### Allele-specific expression analysis

Differential allele-specific expression was tested for two SNPs within the transcripts of each of the genes *CYP21 *and *HSD3B*. The *CYP21 *SNPs were located in exon 8 and 9 and the *HSD3B *SNPs were located in the 5'UTR and in exon 2 (all assays shown in Additional files 1 and 2). For the candidate gene *CYB5A *one SNP in the 5'UTR was tested for differential expression. Additionally, an assay was designed for differential transcription profiling of the *CYB5A *isoforms 1 and 2.

### Genotyping

SNPs were genotyped using matrix-assisted laser desorption/ionisation time-of-flight mass spectroscopy (MALDI-TOF MS) assays. Multiplex assays for use in the Sequenom MassARRAY system were designed using MassARRAY Assay Design software v3.0 (Sequenom, San Diego, USA). Primers for the genotyping are shown in Table 5. Genotyping was done by the IPLEX protocol using manufacturer's instructions (for complete details see iPLEX Application Note, Sequenom, San Diego). The MassARRAY Typer software was used for automated genotype calling.

**Table 5:** Primers used for analyzing SNP's in the candidate genes *CYB5A*, *HSD3B*, *CYP11A1 *and *NCOA4*.

SNP	SNP-Localization	Forward primer	Reverse primer
CYB5A_-8(5'UTR)	-8, 5'UTR	CTCTGTTCCGCTCATCTCTG	ATACTTCACGGCTTTGTCGG
HSD3B_-15(5'UTR)	-15, 5'UTR	TCCCCAGTGTTTTCTGGTTC	CCATCCAGCCATTGCTAAAC
HSD3B_271-exon2	271, exon2	TCATCCACACTGCCTCTATC	TTGACCTTCATGACGGTCTC
CYP11A1_150-exon1	150, exon1	TGCATCTCCACTAAAACCCC	ACGGTACAGGTTAATCCAGC
NCOA4_3'UTR	3' UTR	TGCAGTCCCAGTGTCATTAC	GTTCTAAATGGTATCTGGGG

### Statistical analysis

#### Gene expression

Gene expression was quantified using the MassARRAY QGE software v3.4 (SEQUENOM, San Diego, USA) and TITAN version (1.0-13) [63] that runs in the R statistical environment. Titration of competitor concentration was used to determine the competitor concentration at which cDNA and competitor amplify equally well (EC50). The relative amounts of cDNA and competitor at each titration point were estimated by MassARRAY QGE software (Sequenom, San Diego, USA), using the mass spectra obtained. According to the Sequenom terminology the mass spectra are referred to as allele frequencies based on the calculation of the peak area ratios between extension products within each assay. The sum of all allele frequencies is equal to 1.0 for each assay [64]. For ordinary gene expression there is one frequency for the cDNA and one for the competitor. When measuring allele-specific expression, there is one cDNA frequency per allele, and one for the competitor. For the assays designed for detection of differential allele expression, the expression levels of the alleles were summed before estimation of the total gene expression level of the gene (for the assays designed for estimation of allele expression, see below). The raw data from the Genotype Analyzer Software (Sequenom) was imported into R, where the data was preprocessed in order to remove bad data points (i.e. when no signal was detected for neither cDNA nor competitor), and the median frequency of the printing replicates were calculated. To identify differentially expressed genes, the preprocessed data were analyzed using TITAN. In TITAN the frequencies were first transformed using a log transformation (y = log_10_(f/1-f)), in order to obtain a linear relationship between frequencies and the competitor concentration (log10 scale). After that, a linear model was fitted per gene using the log10 concentration of the competitor as x and high/low androstenone levels as covariates. For each treatment, the model is interpolated in order to find the concentration where the amounts of cDNA and competitor are identical. Log fold changes are calculated as the difference between high and low androstenone on the log scale. The housekeeping gene (*HPRT*) was used for normalization. In the analysis using TITAN, default values of linear least squares polynomial regression and 4000 bootstrap replicates were used. Based on the bootstrap replicates, confidence intervals and p-values for the fold changes were calculated. The threshold for significance was set at P < 0.05. Details about the TITAN software are available from http://www.well.ox.ac.uk/~tprice/titan.

#### Allele-specific expression

The assays for allele-specific expression of SNPs in *CYB5A *and *HSD3B *were designed to amplify transcripts of the two alleles as well as the competitor. The frequencies of the two alleles were summarized in order to get total cDNA and thus total gene expression as described above. The relative expression of allele 1 were determined as the average ratio f_1_/(f_1_+ f_2_) across the whole titration range, where f_1 _and f_2 _are the frequencies of allele 1 and 2, respectively. We used the average ratio across all titration points, since it is reasonable to assume that this ratio is constant across the titration range. In order to find whether the allele expressions were differentially expressed, the general linear model (GLM) procedure of the Statistical Analysis System (SAS) Version 9.1.3 [65] was used. Treatment (high/low androstenone) and alleles were included in the analyses as fixed effects. Results were considered to be significant at P < 0.05.

#### Association study

Associations between androstenone and the four candidate genes *HSD3B, CYP11A1*, *CYB5A *and *NCOA4 *were evaluated using the GLM procedure of SAS Version 9.1 [65]. Models were fitted to identify other significant environmental and genetic effects apart from the genotypes, by elimination of non-significant effects. Levels of androstenone in fat were log-transformed to normalize the distribution of observed values. Analyses were carried out separately for the two populations using the following statistical model:

where Y_ijkl _is ln(ppm levels of androstenone in adipose tissue) of animal j, offspring of sire i; gene_j _is the fixed effect of the candidate gene genotype; hys_k _is the fixed effect of herd/year/season, and bulbo_l _is the random effect of *glandula bulbo urethralis*. Sire was included as fixed effect in the model to ensure that the genotype effects were not confounded with selection in the sires. The length of *glandula bulbo urethralis *is taken into account because it is highly correlated with the level of sexual maturation in boars [66]. Least squares means were estimated for each genotype and overall F tests were used to determine level of significance. Back-transformed least-squares mean without further corrections are presented in Table 4, thus giving the medians in the original skewed distributions. Results were considered to be significant at P < 0.05. Standard errors are supplied in the Table 4. Furthermore, a chi-square test was conducted to test whether any of the SNPs were diverging from Hardy-Weinberg equilibrium.

## Authors' contributions

EG conducted the molecular work, performed statistical analyses and drafted the manuscript. IB did statistical supervision and carried out the programming involved. MM was involved in the molecular work. PO carried out the assay design for gene- and allele expression analyses. SL was involved in planning the project, provided laboratory facilities and took part in writing the paper. All authors read and approved the final manuscript.

## Supplementary Material

Additional file 1 Primer sequences for quantitative gene expression analysis (rcPCR). Click here for file

Additional file 2 Competitor sequences for quantitative gene expression analysis (rcPCR). Click here for file
