# Supplementary material for: Transcript profiling of candidate genes in testis of pigs exhibiting large differences in androstenone levels
Source: BMC Genet. 2010 Jan 25;11:4. doi: 10.1186/1471-2156-11-4 (PMC2823645; doi:10.1186/1471-2156-11-4)
Supplement: Additional file 1 — Primer sequences for quantitative gene expression analysis (rcPCR). [file 1471-2156-11-4-S1.DOC]

**Additional file 1.** Primer sequences for quantitative gene expression analysis (rcPCR).

| Gene name | Accession | Forward primer | Reverse primer | Extension primer |
| --- | --- | --- | --- | --- |
| AKR1C4 | NM_001038626 | AGCTGGAGATGATCCTGAAC | CATTCCACCTGGTTGCAGAC | AGACGGGCTTGTACT |
| CYB5A_8(5’UTR) | AF016388 | CTCTGTTCCGCTCATCTCTG | ATACTTCACGGCTTTGTCGG | gGCAGGGCCTGAGGTTCGCCGC |
| CYB5A_iso1-2 | AF016388 | AGATCAAAGATTGCCAAGCC | GTCCACCAGCTGGAATTAGA | tgaCAAAGATTGCCAAGCCTTCGGAA |
| CYP11A1 | NM_214427 | GGAAATGACAAGCTGCTCTC | ATGGATGTCGTGTCTACACC | CGGTAACATTGGCCTTA |
| CYP17A1 | M63507 | TTCGATTCCGGCCTGTGTCC | ATTCGCCAATGCTGGAGTCAATG | cTGTCCCCTACGCTCA |
| CYP19A2 | NM_214430 | GCTGTTTCTCATTGCAAAGC | GTCTCTTTCACCAATAACAGTCT | ACTATTGCCTCTTCAACC |
| CYP21_exon8 | NM_214433 | ACCCTGAGATTCAGTGGCG | ACGGTCCTTGTATGGGACTC | cCCCGCAGCTCCAGGGCCCAGCTC |
| CYP21_exon9 | NM_214433 | CTAGCAGCATCTTCGGCTAC | AGGTGGGCACCTTGGAGGTT | CTAGCAGCATCTTCGGCTACGACATCC |
| DHRS4 | AB062757 | AGCTGGCCCCAAGGAACATT | TTCAGCCAGGTGTTGTGGATGGA | CCAAGGAACATTAGGGTG |
| FTL | AY610290 | ACTTGGAGAAGGGACTGAAC | AAGTCACAGAGGTGGGGGTCTGC | cACCCAGCGCATGCAGATCC |
| HPRT | AY609416 | CTGGCAAAACAATGCAAACC | TTTCACCAGCAAGCTTGCAACCT | GCAAACCTTGCTTTCC |
| HSD17B4 | X78201 | CCAAGAAACTGGAGACACTG | AGATCTCCACCCTCAGATGGTAT | gAGTATCAGATGTTGGCACA |
| HSD3B_exon2 | AF232699 | GCCTCTATCATCGACGTG | GCTGGGTACCTTTCACATTG | aATGACGGTCTCTCGCCCAACGG |
| HSD3B_5’UTR | AF232699 | TCCCCAGTGTTTTCTGGTTC | CCATCCAGCCATTGCTAAAC | gGGTTCCTGGCAAGTATTTCTCGG |
| NCOA4 | DV227159 | TGAAGATTCGACTGTCCTGC | TCAGGAATTTGGATGGTCTTGAG | gGAAACTGACACAACTGCTCT |
| PGRMC1 | X99714 | TGACCTTTCTGACCTCACTC | ATGGTACTTGAAAGTGAACTGAG | CCAGTCATTCAGGGTCTCC |
| SMPD1 | BP435032 | ATATCATCGGCCGTATTCCC | CTCGTACCTGTTCACGATTC | gCAGGGCACTGCCTGAAG |
| STAR | NM213755 | TTCCGTGTGTGTGCTGGCTG | CGTGCTCAGCTCTGATGACCCCC | tCTCAGGCATCTCTCCAAAGTC |
| SULT2A1 | DQ172907 | CCAAGGAAATGTGCCCTATGGAT | GTATCAGGACGTTCTCCTTG | tACATTCGTGGCTGGT |
