# Supplementary material for: Transcript profiling of candidate genes in testis of pigs exhibiting large differences in androstenone levels
Source: BMC Genet. 2010 Jan 25;11:4. doi: 10.1186/1471-2156-11-4 (PMC2823645; doi:10.1186/1471-2156-11-4)
Supplement: Additional file 2 — Competitor sequences for quantitative gene expression analysis (rcPCR). [file 1471-2156-11-4-S2.DOC]

**Additional file 2. Competitor sequences for quantitative gene expression analysis (rcPCR)^a^**

| Gene name | Competitor sequence |
| --- | --- |
| AKR1C4 | CATTCCACCTGGTTGCAGACGGGCTTGTACT**c**GAGCCCTGGCTTGTTCAGGATCATCTCCAGCT |
| CYB5A_-8(5’UTR) | ATACTTCACGGCTTTGTCGGACTGTTCGGCCATTTCGTAA**g**GCGGCGAACCTCAGGCCCTGCTGACAGCGGCAGAGATGAGCGGAACAGAG |
| CYB5A_iso1-2 | GTCCACCAGCTGGAATTAGATTCAACAGTGGTAATAAGAG**c**TTCCGAAGGCTTGGCAATCTTTGATCTG |
| CYP11A1 | ATGGATGTCGTGTCTACACCCCCTGCCAGCATCTCGGTAACATTGGCCTTA**t**CATCTTCTGAGAGCAGCTTGTCATTTCC |
| CYP17 | ATTCGCCAATGCTGGAGTCAATGATAGCCCTGTGGGGG**g**TGAGCGTAGGGGACACAGGCCGGAATCGAA |
| CYP19A2 | GTCTCTTTCACCAATAACAGTCTGGATTTCCTTCACTATTGCCTCTTCAACC**c**GGGGGTGCTTTGCAATGAGAAACAGC |
| CYP21_exon8 | ACGGTCCTTGTATGGGACTCGGGAGCCCGCAGCTCCAGGGCCCAGCTC**t**CGATCTAACTCCTCCTGCAGCCGCCACTGAATCTCAGGGT |
| CYP21_exon9 | AGGTGGGCACCTTGGAGGTTGGGGATGACAACGGTGCCCTCA**c**GGATGTCGTAGCCGAAGATGCTGCTAG |
| DHRS4 | TCCATCCACAACACCTGGCTGAAGTTAGTCTTGATGAGTCCAGGCGCCAGGCAGT**a**CACCCTAATGTTCCTTGGGGCCAGCT |
| FTL | AAGTCACAGAGGTGGGGGTCTGCGCGGGCAGAACCCAGCGCATGCAGATCC**t**CAAGAGCCTGGTTCAGTCCCTTCTCCAAGT |
| HPRT | TTTCACCAGCAAGCTTGCAACCTTGACCATCTTTGGATTATGCTGCTTGACCA**g**GGAAAGCAAGGTTTGCATTGTTTTGCCAG |
| HSD17B4 | AGATCTCCACCCTCAGATGGTATCTTAGCTAAAGTATCAGATGTTGGCACA**t**GATCCACGTATGCATTTGAAATGACAGTGTCTCCAGTTTCTTGG |
| HSD3B_exon2 | GCTGGGTACCTTTCACATTGACCTTCATGACGGTCTCTCGCCCAACGG**g**GTTGACCACGTCGATGATAGAGGC |
| HSD3B_5’UTR | CCATCCAGCCATTGCTAAACCTGGGC**c**CCGAGAAATACTTGCCAGGAACCAGAAAACACTGGGGA |
| NCOA4 | TCAGGAATTTGGATGGTCTTGAGGGACCCAAATGTGGTGATGGCCTGGCG**a**AGAGCAGTTGTGTCAGTTTCAAAAAGCAGGACAGTCGAATCTTCA |
| PGRMC1 | ATGGTACTTGAAAGTGAACTGAGAGTCCCAGTCATTCAGGGTCTCC**c**GCTGGGCAGGAGTGAGGTCAGAAAGGTCA |
| SMPD1 | CTCGTACCTGTTCACGATTCGGTAATAATTCCAGCTCCAGC**a**CTTCAGGCAGTGCCCTGGGGGAATACGGCCGATGATAT |
| STAR | CGTGCTCAGCTCTGATGACCCCCTTCTGCTCAGGCATCTCTCCAAAGTC**c**GTGGCCATGCCAGCCAGCACACACACGGAA |
| SULT2A1 | GTATCAGGACGTTCTCCTTGTCTCGCATGGGC**g**ACCAGCCACGAATGTGGTCAAACCATGATCCATAGGGCACATTTCCTTGG |

^a^Artificial mutations introduced in the competitor sequences are indicated with grey lower-case letters. Accession number as in Table 1.
